# Long Term Complete Remission in Advanced Gastric Adenocarcinoma With Docetaxel, Oxaliplatin and Capecitabine Combination Regimen

**DOI:** 10.4021/wjon469w

**Published:** 2012-07-05

**Authors:** Gaurav Goel

**Affiliations:** Department of Internal Medicine, Montefiore Medical Center, 600 E 233rd Street, Bronx, New York, 10470, USA. Email: gaugoel@hotmail.com

**Keywords:** Complete remission, Gastric cancer, Docetaxel, Oxaliplatin, Capecitabine

## Abstract

Gastrointestinal tract cancers are highly lethal malignancies with early metastatic dissemination. Chemotherapy is therefore of crucial importance in advanced cancer for obtaining palliation of symptoms and improving survival. However, there is no universal standard chemotherapy regimen for the treatment of gastroesophageal cancer. Also, complete remission is an uncommon outcome in advanced gastroesophageal cancers treated with chemotherapy; and where encountered, the response is not sustained. We present a 65 year-old patient who presented with advanced gastric adenocarcinoma. She had histopathologically proven liver metastases. She was treated with chemotherapy comprising of six cycles of Docetaxel, Oxaliplatin and Capecitabine (DOX). She showed complete remission with disappearance of local as well as liver lesions, and achieved disease free status for 9 months. This is the first known sustained Complete Response in a case of advanced gastric carcinoma with DOX combination regimen.

## Introduction

Cancers of the upper gastrointestinal (GI) tract are highly lethal malignancies with propensity for early metastatic dissemination [1]. Gastric cancer is the fourth most commonly diagnosed cancer and the second leading cause of cancer death worldwide [2]. Chemotherapy is therefore of crucial importance in advanced gastroesophageal cancer patients in order to obtain palliation of symptoms and improve survival. Single agents with activity in advanced gastric cancer include 5-fluorouracil (5FU), cisplatin, the anthracyclines- doxorubicin and epirubicin, mitomycin C, etoposide; all associated with modest response rates of short duration and infrequent complete responses [3]. Various combinations of these agents have been tried to improve upon these results. However, no single regimen has proclaimed precedence over the others, and currently there is no universal standard regimen for the treatment of gastroesophageal cancer [4]. Data from several phase II trials suggest that complete remission (CR) is an uncommon outcome in advanced gastroesophageal cancers treated with chemotherapy. The V325 phase III trial of docetaxel, cisplatin and 5FU (DCF) in gastric cancer showed CR in 4 (2%) patients (n = 221) [5]. Moreover, in cases where CR is encountered, the response is not sustained. Thus, new treatment protocols are warranted to achieve better disease control. We document a sustained complete remission with docetaxel, oxaliplatin and capecitabine (DOX) combination regimen in a case of advanced gastric carcinoma.

## Case Report

**Figure 1:**
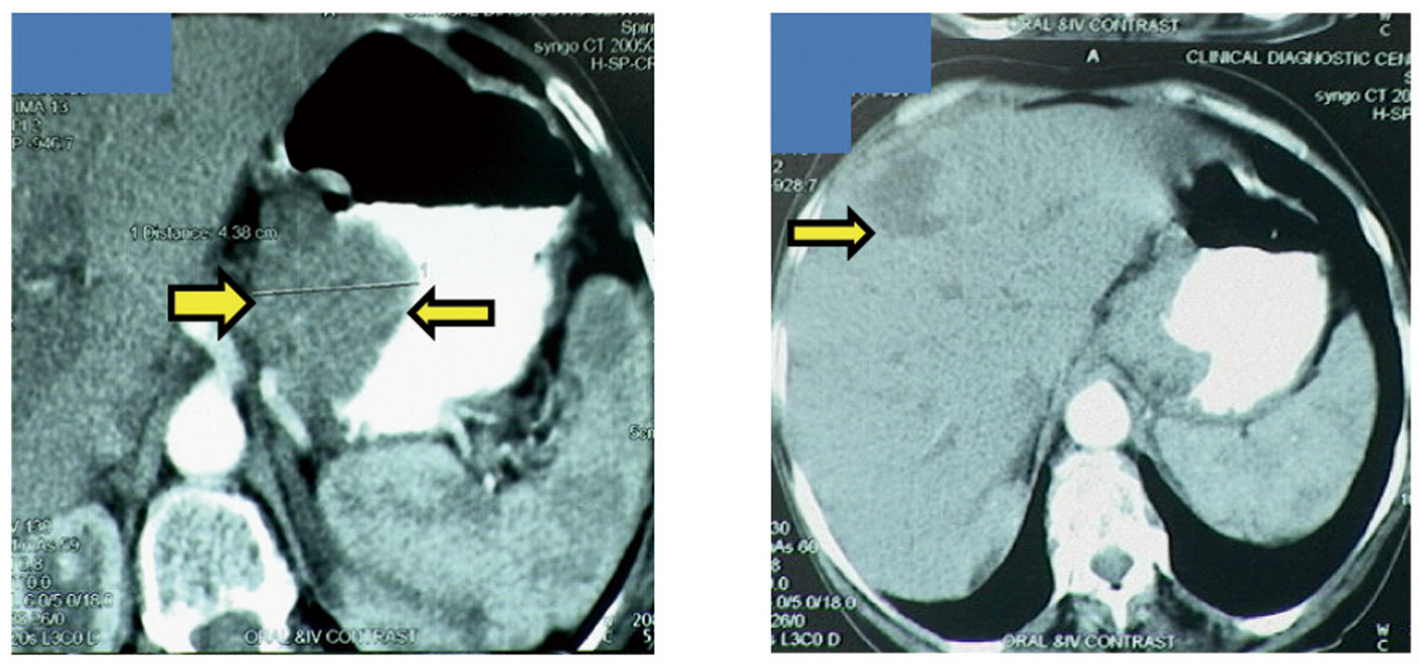
Disease status at the time of diagnosis. Gastric cancer with gastric wall thickness of 44 mm (left), Liver metastases (right).

**Figure 2:**
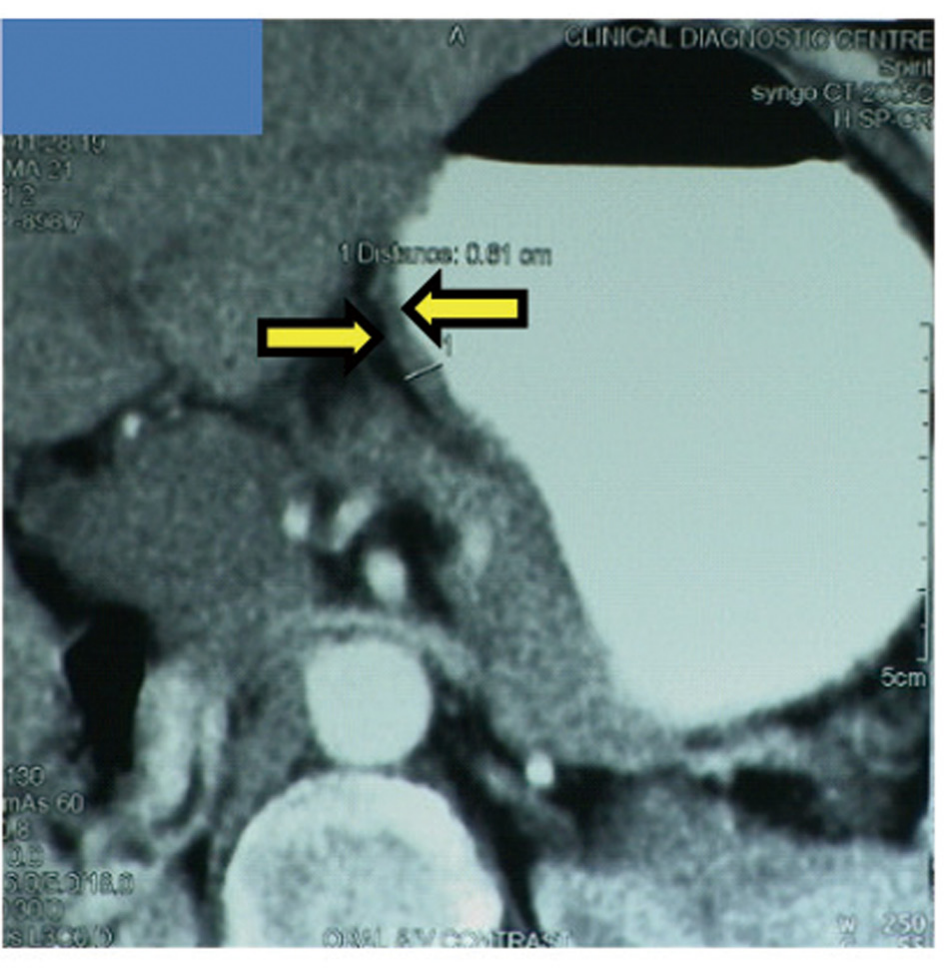
Disease status after 6 cycles of chemotherapy. Gastric cancer with gastric wall thickness of 8 mm.

**Figure 3:**
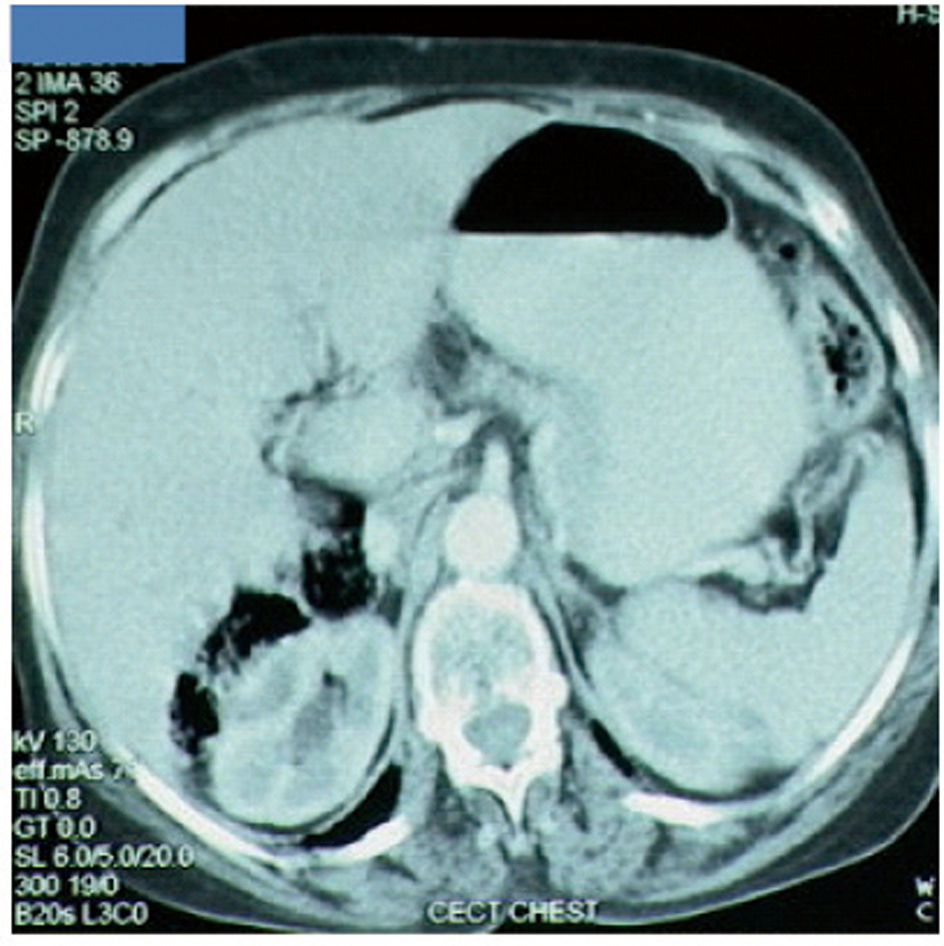
Disease status at 9 months after diagnosis. Complete disappearance of gastric and liver lesions.

A 65-year-old postmenopausal woman presented in May 2007 with vomiting, early satiety and 20 lbs weight loss over last 2 months. There was no history of associated fever, cough, bleeding from any site, and the family history was also insignificant. On examination, pallor was present. However, no icterus, edema or lymphadenopathy was noted. Detailed systemic examination revealed no abnormality. Investigations revealed mild anemia (Hb 9.8 g/dL) and elevated alkaline phosphatase levels (310 U/L). Esophagogastroduodenoscopy (EGD) showed large ulcerated lesion at the fundus, extending into the esophagus. CECT abdomen showed mass lesion in gastric cardia extending to involve the gastroesophageal junction and fundus, with compression of fat planes and wall thickness of 44 mm. There were multiple space occupying lesions (SOLs) in the right lobe of liver, the largest measuring 4.3 x 3.1 cm (Fig. 1). Tissue specimens from endoscopic biopsy (gastric region) and CT guided FNAC of liver showed moderately differentiated adenocarcinoma. Hence, a diagnosis of gastric carcinoma with liver metastases was made. Tumor markers - CEA and CA 72.4 levels were 1.7ng/mL and 0.99 U/mL respectively. The patient was then started on chemotherapy regimen consisting of docetaxel, oxaliplatin and capecitabine (DOX). Docetaxel was administered as 25 mg/m^2^ followed by oxaliplatin 50 mg/m^2^ on days 1 and 8, with capecitabine 625 mg/m^2^ BID from days 1 to 14, in 21-day cycles. The protocol was well tolerated by the patient except for grade 2 hematologic toxicity, which responded to blood product transfusions, erythropoietin and G-CSF. Patient also experienced diarrhea, managed with supportive therapy. A thorough workup done was after 3 and 6 cycles of chemotherapy to evaluate the response. Investigations at the end of 6 cycles of chemotherapy (November 2007) showed decreased gastric lesion, with a wall thickness of 8 mm on CT abdomen (Fig. 2); the particular liver lesion also decreased in size to 1.3 x 1.1 cm. EGD revealed few small erosions in fundus near the gastroesophageal junction, no ulcer or growth was seen. CT abdomen done 3 months later (February 2008) showed complete absence of gastric and liver lesions (Fig. 3). This patient responded dramatically to the chemotherapy protocol, as evidenced by > 50% decrease in the size of gastric and liver lesions after 6 cycles of chemotherapy, and complete disappearance of the lesions after further 3 months. Hence the patient’s clinical response to chemotherapy was labeled as CR. The patient continued to maintain CR for about 9 months, since the initial response.

## Discussion

Palliative systemic chemotherapy is more beneficial as compared to supportive care alone for patients with advanced gastric cancer [6]. Cytotoxic chemotherapy can provide symptom palliation, improve quality of life, and prolong survival in these patients. Meta-analysis has shown that combination chemotherapy regimens provide higher response rates including CR than do single agents [7]. Despite a large number of randomized trials, there is no consensus as to the best regimen. The combination of cisplatin plus 5-FU has been one of the most commonly used regimens in both metastatic and localized gastric cancer due to its activity and well-established toxicity profile. The V325 trial showed that addition of docetaxel to cisplatin-5FU resulted in increases in the CR [5]. It established a role for docetaxel in the treatment of advanced gastroesophageal cancer. However, DCF has significant toxicity, tolerability issues and needs ambulatory infusion pump. Cunningham et al. in a randomized, phase 3 study showed that for advanced esophagogastric cancer, oral capecitabine and oxaliplatin are non-inferior to infused fluorouracil and cisplatin respectively [8].

With this background, we modified the efficacious, albeit toxic DCF regimen, by replacing cisplatin with oxaliplatin and 5FU with capecitabine, to make it more tolerable and easier to administer while maintaining efficacy. The rationale for combining docetaxel with capecitabine is the synergism between the two, as docetaxel upregulates thymidine phosphorylase enzyme in the tumor cells. This enzyme is responsible for conversion of capecitabine to its active metabolite, 5-FU that kills cancer cells [9]. DOX regimen therefore utilizes the promising activity of all 3 agents against gastroesophageal cancer. The Brown University Oncology Group has conducted a phase I trial to study this combination of DOX in patients with metastatic esophageal and gastric cancer [10]. Goel et al. have studied the DOX combination regimen in this patient population and demonstrated CR in 3 (14%) patients (n = 21) [11]. Our patient achieved CR post 6-cycles and continued to maintain remission for about 9 months. To conclude, the regimen of docetaxel, oxaliplatin, and capecitabine is a promising, well tolerated chemotherapy regimen in advanced gastroesophageal cancer, and merits further evaluation.
